# Night Sleep and Parental Bedtime Practices in Low-Risk Preterm and Full-Term Late Talkers

**DOI:** 10.3390/children9121813

**Published:** 2022-11-24

**Authors:** Alessandra Sansavini, Martina Riva, Mariagrazia Zuccarini, Arianna Aceti, Luigi Corvaglia, Anat Scher, Annalisa Guarini

**Affiliations:** 1 Department of Psychology “Renzo Canestrari”, University of Bologna, Viale Berti Pichat 5, 40127 Bologna, Italy; 2 Department of Education Studies “Giovanni Maria Bertin”, University of Bologna, Via Filippo Re 6, 40126 Bologna, Italy; 3 Neonatal Intensive Care Unit, IRCCS Azienda Ospedaliero-Universitaria Bologna, Via Massarenti 9, 40138 Bologna, Italy; 4 Department of Medical and Surgical Sciences, University of Bologna, Via Massarenti 9, 40138 Bologna, Italy; 5 Department of Counseling and Human Development, University of Haifa, Abba Khoushy Ave 199, Haifa 3498838, Israel

**Keywords:** night sleep, parental bedtime practices, late talkers, risk factors, low-risk preterm birth, birth order, maternal education, parenting stress

## Abstract

Night sleep and parental bedtime practices have rarely been investigated in late talkers. This study aimed to explore: night sleep, parental bedtime practices, and their associations in late talkers as well as individual, socio-demographic, and socio-relational factors affecting them. Parents of 47 30-month-old late talkers, born low-risk preterm (*n* = 24) or full-term (*n* = 23), with an expressive vocabulary size ≤10th percentile measured by the MacArthur-Bates Communicative Development Inventory Words and Sentences, and normal cognitive abilities measured by the Bayley Scales, completed the Infant Sleep Questionnaire, the Parental Interactive Bedtime Behaviour Scale, and the Parenting Stress Index Short Form. Results showed slight settling difficulties, night wakings, and frequent co-sleeping in late talkers. Encouraging autonomy practices were frequently used by parents, rather than active physical comforting ones. Recurrent settling difficulties were reported by parents who often applied encouraging autonomy practices, whereas greater night waking problems and frequent co-sleeping were reported by parents who often left their child crying. Low-risk preterm birth and mother’s parenting stress predicted total sleep difficulties and night wakings; first-born, high maternal education level and mother’s parenting stress predicted settling difficulties; mother’s parenting stress was the only predictor for co-sleeping and leaving to cry. These findings have relevant implications for improving late talkers’ night sleep and their parents’ bedtime practices.

## 1. Introduction

Sleep is essential for healthy child development and its characteristics are related to cognitive, linguistic, behavioral, and motor development from very early in life [1,2,3,4,5,6,7]. Sleep patterns, like daytime and night duration, efficiency, and rhythm, change with development. Concerning typically developing children, daytime sleep duration decreases, whereas night sleep duration increases from birth onward [7,8,9,10], with a more stable consolidation occurring during late adolescence [11,12]. In addition, sleep efficiency, i.e., the ratio between minutes of sleep and sleep duration (from falling asleep until morning awakening), increases with age as the number and duration of night wakings decrease [8,9,10]. Wide interindividual variability in sleep patterns has been observed in infancy, with different sleep duration trajectories depending on individual and environmental factors [12,13,14,15].

### 1.1. Night Sleep Characteristics in Atypical Development

Sleep organization and consolidation processes follow different trajectories in atypically developing children compared to typically developing ones. According to Scher et al. [16], night sleep difficulties are more frequent in infants with a higher risk for developmental delays in the first year of life compared to infants at no-risk or low-risk. D’Souza and colleagues [17] reported more night sleep disruptions in children with neurodevelopmental disorders (e.g., Down syndrome, Fragile X syndrome, Williams syndrome) than in typically developing children, explained by reduced sleep efficiency.

Little is known about night sleep in late talkers, i.e., 2–3-year-old children with an expressive vocabulary size ≤10th percentile assessed by parental questionnaires, such as the MacArthur–Bates Communicative Development Inventories (MB-CDI) [18], and no neurological disorders, intellectual disabilities, or sensory impairments [19,20,21,22,23]. One study found no significant differences in night sleep features between late talkers and typically developing children by using parental questionnaires, including a few items on sleep features [23]. By contrast, other studies revealed an association between night sleep characteristics and linguistic skills. Indeed, children with shorter night sleep duration had lower linguistic scores compared to children with longer sleep duration [4]. In addition, Dionne et al. [1] and Hernandez-Reif and Gungordu [24] respectively observed that aspects of night sleep quality, such as greater sleep consolidation at six and 18 months and optimal sleep at eight months, predicted better linguistic skills at 14–18, 30, and 60 months.

### 1.2. Parental Bedtime Practices

Infant sleep can be affected by parents’ interactive behaviors and bedtime practices which, in turn, may be affected by infant’s characteristics, such as age, personality, developmental features, or sleep patterns that can increase parental stress [25].

Parental bedtime practices have been grouped into five main categories: active physical comforting (e.g., “cuddling or rocking in arms”), encouraging autonomy (e.g., “offering a special toy/cloth”), settling by movement (e.g., “walking in pram or buggy”), passive physical comforting (e.g., “lying with child next to their cot”), and social comforting (e.g., “reading a story”) [26]. The use of several types of parental bedtime practices varies according to children’s needs and age: active physical comforting practices usually decrease over time, whereas encouraging autonomy practices increase [9]. The use of bedtime practices depends as well on parents’ culture [25,27]. In their review, Sadeh and colleagues [25] highlighted a strong influence of socio-cultural norms, values, and ethnicity on parental bedtime practices, expectations regarding infant sleep, and sleep behaviors perceived as problematic. For example, co-sleeping is the most common bedtime parental practice in the majority of cultures, yet it is considered a controversial practice in Western industrialized societies [28]. In the first three years of life, physical comforting is associated with sleep problems, especially if physical comforting practices are not sufficiently balanced by encouraging autonomy practices, which have been found as beneficial to sleep in studies conducted with UK and US samples [9,26]. In addition, the regularity of parental bedtime practices is a predictor of good sleep quality [9]. The above findings on the links between bedtime practices and children’s sleep were derived from data obtained mainly in Western societies. It is essential to keep in mind that cultural context not only shapes parenting practices [29], but broadly defines what is considered normal sleep versus problematic patterns [30]. Sleep-related “problems”, such as difficulties falling asleep alone or waking at night and seeking parental attention, are based on culturally constructed definitions and expectations and are not necessarily rooted in sleep biology [30,31]. Cultural norms and expectations define what is considered as a desired sleep pattern, and in turn, influence the parenting practices that promote such patterns [32]. Importantly, these cultural variations also transpire in sleep duration and quality. For example, Dutch compared to US infants sleep longer and spend more time in quiet as compared to active sleep [32]. Moreover, Hale et al. [27] found that ethnicity is a predictor of bedtime routines. They observed that Caucasian caregivers are more likely to use bedtime routines compared to non-Caucasians ones.

Culturally specific parental bedtime practices can affect not only infant sleep patterns, but also infant health and development, parent–infant attachment, and family functioning, by reducing maternal stress and increasing the perception of parental self-efficacy [33]. For example, among encouraging autonomy practices, social and verbal bedtime interactions that enhance self-regulation [34,35] have been linked to better sleep quality and longer night sleep duration [26]. Noteworthy is that these soothing parental practices are associated with better health and higher developmental outcomes [27]. Hale et al. [27] found enhanced receptive and expressive language skills in five-year-old children whose parents had employed verbal bedtime strategies at three years of age, compared to those whose parents employed non-verbal strategies at the same age. Additionally, Williams and Horst [36] highlighted the benefits of shared storybook reading before falling asleep for word acquisition, especially if the same story was read more than once, in three-year-old children. Some studies have also shown that sleep promotes acquisition of lexical, semantic, and grammatical skills if it has adequate duration and if the child’s falling asleep occurs close to verbal stimuli exposure [37,38,39,40].

### 1.3. Risk Factors Affecting Night Sleep Characteristics and Parental Bedtime Practices

Night and daytime sleep duration and sleep quality (e.g., difficulties to fall asleep and night waking) can be affected by individual factors, such as child age, functional organization occurring in the central nervous system, and circadian rhythm consolidation [9,10,41], as well as by environmental factors, such as parents’ cultural [42] and socio-demographic characteristics, psychological functioning, and relationship with the child [9].

Concerning individual factors, in the first six years of life, shorter night sleep durations were related to first-born status, probably because of higher parental stress or interventions, and more frequently to male than female gender [13,43]. Furthermore, significant alterations in sleep patterns were found in children born preterm, before 37 weeks of gestation, and particularly in those with higher levels of neonatal immaturity, even if there is no concordance in the literature about preterm birth being a risk factor for the emergence of sleep problems [12,44,45,46,47].

With regard to socio-demographic characteristics, shorter early child night sleep durations were found to be associated with older maternal age (>35 years old), lower maternal education [48], and a higher number of siblings in the family [13,43].

Concerning parents’, and particularly mothers’, psychological functioning and relationship with the child, night sleep duration and quality were affected by anxiety, stress, depression, beliefs, quality of parent-child interaction, and attachment in early childhood [33,49,50,51,52,53,54,55,56] (in these studies children were aged between two and 12 months [50,52,54,55,56], 1–2-years-old [53], 2–3-years-old [51], 2–36 months [49], and from birth to five years old [33]). In particular, length of night wakings, as reported by parents, was negatively related to attachment security, and re-settling after sleep awakening was linked to the quality of the mother-infant relationship at 12 months [55]. In addition, Bacaro and colleagues [49] found lower infant night sleep quality and shorter sleep duration as well as higher stress levels in parents of children aged 2–36 months with sleep problems, compared to parents of children with no sleep problems. Similarly, de Stasio and colleagues [51] found a significant association between the number of night wakings, difficulty in falling asleep, and parental stress in children aged 2–3-years-old.

Parental bedtime practices are also affected by demographics, familial environmental, and cultural factors. For example, increased use of parental bedtime practices to settle preschoolers to sleep was related to higher parental level of education, older maternal age, and more bedrooms in the home; by contrast, bedtime involvement to settle the child to sleep was less prevalent in families with a greater number of children [27,57]. In addition, bedtime practices were more likely used by Caucasian caregivers compared to non-Caucasian ones, whereas co-sleeping was more frequently used in Asian countries compared to Caucasian ones [42,58,59].

### 1.4. The Present Study

Because of the scarcity of studies in the literature about night sleep characteristics and bedtime parental practices in late talkers, the present study aimed to describe perceived night sleep characteristics of late talkers, i.e., settling difficulties, night wakings, and co-sleeping, as well as bedtime practices used by late talkers’ parents. We also aimed to describe the associations between perceived night sleep characteristics and parental bedtime practices in late talkers. Regarding the description of perceived night sleep characteristics and parental bedtime practices and their relationships, this study can be considered explorative because it is the first one, to our knowledge, that investigates these aspects of late talkers. Based on previous research on typically developing children, we hypothesized that practices to encourage autonomous falling asleep are more common at 30 months than active physical comforting ones, more frequently used in younger infants [9]. Consistent with previous research [9,25,26,27], we also expected associations between perceived night sleep difficulties and the use of parental bedtime practices.

A further aim was to investigate how individual, socio-demographic, and socio-relational variables, i.e., birth condition, gender, birth order, maternal age, maternal education, and maternal stress concerning the child, were associated with perceived sleep difficulties and parental bedtime practices in late talkers. Based on previous research [13,33,43,48,49,51,57], we expected a link between perceived sleep difficulties and preterm birth, first-born status, older maternal age, low maternal educational level, and higher mother’s parenting stress. Regarding parental bedtime practices, we hypothesized that their use would be associated with mother’s parenting stress levels and that encouraging autonomy bedtime practices would be more frequently applied by older mothers, mothers with higher educational levels, and mothers of first-borns.

## 2. Materials and Methods

### 2.1. Participants

Participants were recruited within a screening project (for details about the screening, see Sansavini, Zuccarini et al., 2021 [60]), aimed at identifying language profiles among 30-month-old low-risk preterm children (i.e., with a gestational age < 37 weeks and absence of severe perinatal complications) and a comparable group of full-term children (i.e., with a gestational age ≥ 37 weeks), all born at the IRCCS AOU Bologna, Italy. Recruitment criteria were the following: (a) being monolingual or mainly exposed (>65% of daily exposure) to the Italian language from birth onward; and (b) not having a major cerebral damage (i.e., hydrocephalus, periventricular leukomalacia) and/or congenital malformations, visual (i.e., blindness, retinopathy of prematurity), auditory (i.e., deafness), or motor impairments, or severe cognitive deficits (identified by a Bayley-III composite cognitive score < 70). 

Forty-seven children, identified as late talkers (see the Outcome Classification and Procedure paragraph for details), consisting of 24 low-risk preterm children and 23 full-term children, and their parents were included in the sample. The mean age of late talkers at the assessment was 31.33 months (*SD* = 1.49, range = 27.20–34.26). Preterm children’s age was corrected for weeks of prematurity to consider their neurobiological immaturity, as in previous studies [61,62]. Individual, socio-demographic and socio-relational characteristics of participants are described in Table 1. Late talkers had a mean gestational age of 36.50 weeks (*SD* = 3.47, range = 30–41.29) and a mean birth weight of 2631 g (*SD* = 864, range = 1080–4040). Most participants were males (83%). Fourteen children were twins (29.8%) and 22 were born by caesarean delivery (46.8%). The mean length of neonatal hospitalization was 12.15 days (*SD* = 17.67, range = 0–78). Mean mothers’ age was 38.22 years (*SD* = 5.76, range = 25–49) and mean fathers’ age was 40 years (*SD* = 6.15, range = 29–52); the majority of mothers (68.1%) and about half of fathers’ (46.8%) had a high educational level (i.e., >13 years of education). Most of the parents were Italian (85.1% of mothers and 87.2% of fathers).

Concerning birth condition, as expected from the literature [63], low-risk preterm late talkers, compared with those born at term, had a significantly lower gestational age and birth weight, and a significantly higher number of days of hospitalization, caesarean delivery, and twins; the two groups did not differ significantly on any other variable (see Table 1). Only a few low-risk preterm late talkers had neonatal complications: 4 were small for gestational age (i.e., infants with a birthweight <10th percentile for gestational age); 11 had respiratory distress (i.e., acute illness coming on within 4–6 h of delivery, characterized clinically by respiratory rate ≥ 60/min, dyspnea and respiratory distress); 1 had apnea (i.e., more than four episodes of apnea/hour or more than two episodes of apnea/hour if ventilation with a bag and mask was required); 1 had bronchopulmonary dysplasia (i.e., needing both supplemental oxygen for ≥28 days and at 36 weeks of post-conception age). None had intra-ventricular hemorrhage, sepsis, or retinopathy of prematurity. These results were expected because they are in line with data reported in the Global Action Report on Preterm Birth [63].

Concerning late talkers’ individual characteristics, as expected from the literature (for a systematic review see Sansavini, Favilla et al., 2021 [64]), male gender was prevalent (i.e., about 83%) among them. Concerning late talkers’ socio-demographic characteristics, these are in line with those of a larger sample of low-risk preterm and full-term children recruited in the same region (see Sansavini, Zuccarini et al., 2021 [60]).

The study met ethical guidelines for human subject protections, including adherence to the legal requirements of Italy and received formal approval from the Bologna Health Authority’s Independent Ethics Committee (protocol numbers: EM 194-2017__76/2013/U/Sper/AOUBo, EM 193-2018_76/2013/U/Sper/AOUBo, EM1229-2020_76/2013/U/Sper/AOUBo). All parents gave informed written consent for study participation, data analysis, and data publication.

### 2.2. Tools

#### 2.2.1. The Italian Version of the MacArthur Bates Communicative Development Inventories (MB-CDI), Words and Sentences Short-Form

The MacArthur-Bates Communicative Development Inventories (MB-CDI) Words and Sentences Short Form is a parental questionnaire assessing expressive lexical and syntactic skills of children aged 18–36 months. The MB-CDI is a valid and reliable tool for screening programs on late talkers [19,60]. The Italian version of the MB-CDI Words and Sentences Short Form has been validated on 816 Italian children aged 18–36 months, showing a high concurrent validity (Pearson *r* = 0.92) with the Words and Sentences Complete Form [65,66]. For the current study, we used the first part of the questionnaire, concerning word production, that includes a list of 100 words. Parents were asked to indicate the words spontaneously produced by their child. A score of 1 was assigned for each item checked. The total number of produced words indicated the size of word production.

#### 2.2.2. Bayley Scales of Infant Development (BSID-III)

The Bayley Scales of Infant Development (BSID-III) [67] is a standardized test for assessing cognitive, motor and language development in children between 1 and 42 months of age. For the current study, we assessed cognitive and language development. The cognitive scale assesses cognitive skills (i.e., sensorimotor development, exploration and manipulation, concept formation, memory, attentive skills); the language scale includes two subscales assessing receptive (i.e., preverbal and verbal comprehension, social referencing, receptive vocabulary) and expressive skills (i.e., preverbal communication and expressive vocabulary). Cognitive and language standardized scores (composite scores) were used by referring to the Italian normative values (mean of 100 and SD of 15) [68]. The BSID-III is a valid and reliable tool in both research and clinical practice with test–retest reliability ranging from 0.67 to 0.94, internal consistency coefficients (using the split half method) of 0.87–0.93 in the US version and of 0.87–0.94 in the Italian version, and moderate to high correlations with measures of similar domains [67,68], and it has been used in previous studies on late talkers [19,69,70].

#### 2.2.3. Infant Sleep Questionnaire (ISQ)

The Infant Sleep Questionnaire (ISQ) is a parental questionnaire suited to measure perceived sleeping behavior in infants and toddlers [71]. This tool has been validated for 12–18-month-old infants [71]. Recently, it was also adopted for 24-month-old children in a study by Morales-Muñoz and colleagues [72]. Furthermore, a revised version, with an additional item rating intentional co-sleeping, was used in a study on 48–56-month-old children by Aviezer and Scher [73]. In the current study, the questionnaire has been translated into Italian by one of the authors (A.S.) of the manuscript and back translated by a professional translator. In the ISQ questionnaire, parents are asked to describe and rate their child’s sleep behaviors through ten items, each graded on a Likert scale. The following items were used in the current study: ISQ 1 (settling latency; range 0–6; “How long does it take to settle your baby off to sleep on average?”) and ISQ 2 (settling difficulties per week; range 0–7; “How many times a week do you have problems settling him/her on average?”), ISQ 4 (night waking per week; range 0–7; “How many nights a week does your baby wake on average?”), ISQ 5 (night waking per night; range 0–5; “How many times does your baby wake each night and need resettling on average?”) and ISQ 6 (sleep latency after night waking; range 0–6; “If your baby wakes, how long does it take for your baby to go back to sleep on average?”), and ISQ 8 (co-sleeping at night; range 0–7; “How often do you end up taking your child into your bed because your child is upset?”). The questionnaire provides an index of child’s sleep difficulties by computing a total score summing up the scores of items ISQ 1, ISQ 2, ISQ 4, ISQ 5, ISQ 6, and ISQ 8 (*α* = 0.58, [73]; *α* = 0.63, Italian translation). In addition, in the current study, two subscales were obtained: the first subscale, concerning settling difficulties, was obtained by summing up the scores of ISQ 1 and ISQ 2 items (range 0–13); the second subscale, concerning night waking, was computed by summing up the scores of ISQ 4-5-6 items (range 0–18). The co-sleeping item ISQ 8 (range 0–7) was maintained as a separate item and not included in the above two subscales as co-sleeping may be due to multiple factors and not always related to sleep difficulties. The internal validity of the above two subscales was checked (settling: *α* = 0.78; night waking: *α* = 0.52).

The last item, ISQ 10, concerns overall maternal perception about the child’s sleeping difficulties. Mothers were asked to rate whether they judged their child to have or not have sleeping difficulties on a 4-point Likert scale (0 = no problems, mild = 1, moderate = 2, severe problems = 3). According to Morrell [71], in the current study, the criterion absence vs mild/moderate/severe problems was used as maternal criterion of child’s sleep difficulties.

#### 2.2.4. Parental Interactive Bedtime Behaviour Scale (PIBBS)

The Parental Interactive Bedtime Behaviour Scale (PIBBS) is a parental questionnaire assessing behaviors that parents may use for settling their child before sleeping. The PIBBS has been validated for 12–19-month-old-infants [26]. It has also recently been used with parents of children with autism spectrum disorder with a mean age of 39 months, compared to typically developing children with a mean age of 36 months [74]. In the current study, the questionnaire has been translated into Italian by one of the authors (A.S.) of the manuscript and by a professional translator for the back translation method.

The PIBBS is constituted of 17 items exploring the types and frequency of behaviors parents use to settle their child to sleep. Specifically, parents should rate on a five-point Likert scale from never (score = 0) to very often (score = 4) whether they adopted the behavior described in each item for settling the child off to sleep. The internal validity of this questionnaire was computed (*α* = 0.712, [26]; *α* = 0.71, Italian translation).

For the purposes of the current study, we grouped items in two subscales: active physical comforting (items 1, 2, 3, 4, 5, 10, 12, 15, 17; respectively: “stroking part of child or patting”; “cuddling or rocking in arms”; “carrying around the house in arms”; “walking in pram or buggy”; “car riding”; “playing with child”; “giving a feed/drink”; “settling on sofa with parent”; “settling in parent’s bed”) and encouraging autonomy (items 6, 7, 8, 9, 11, 14, 16; respectively: “music tapes or musical toys”; “talking softly to the child”; “singing a lullaby”; “reading a story to the child”; “offering a special toy/cloth”; “standing near cot without picking baby up”; “lying with child next to their cot”). We maintained item 13 (i.e., “leaving to cry”) as a separate item and did not include it in any of the two subscales because it does not involve any intervention from parents. The internal validity of the above two subscales was checked (active physical comforting, *α* = 0.65; encouraging autonomy, *α* = 0.68).

#### 2.2.5. Parenting Stress Index (PSI-4) Short Form

The Parenting Stress Index (PSI-4) Short Form is a parental self-reported questionnaire assessing the extent of stress in the parent-child system. In the present study, we adopted the Italian version [75] of the Parenting Stress Index PSI-4 [76], validated with the Italian population. This tool has been validated for parents of children aged 1 month–10 years old.

The PSI-4 Short Form is comprised of 36 items that are rated on a five-point Likert scale from strongly disagree (score = 1) to strongly agree (score = 5). The items can be grouped into three subscales: Parental Distress (PD) (items 1–12), representing the level of distress experienced by a parent due to factors related to the parental role; Parent–Child Dysfunctional Interaction (PCD-I) (items 13–24), focusing on perceptions of the child as not meeting parental expectations and of interactions with the child that do not reinforce them as parents; and Difficult Child (DC) (items 25–36), defining some characteristics of the child behavior that make him/her easy or difficult to handle. The scores of these domains form a Total Stress scale, in which a higher score corresponds to more elevated levels of perceived stress. The PSI-4 Short Form also includes a Defensive Response scale (DR) (items 1, 2, 3, 7, 8, 9, 11), to check the validity of the protocol, which is useful for assessing the degree to which the parent responds with a tendency to give a more favorable self-image or to minimize problems. The reliability and internal validity of the total scale and the subscales have been provided for the Italian short version [75] (total stress: test-retest = 0.83, *α* = 0.89; parental distress: test-retest = 0.78, *α* = 0.80; parent-child dysfunctional interaction: test-retest = 0.79, *α* = 0.81; difficult child: test-retest = 0.70, *α* = 0.72; defensive response: test-retest = 0.69, *α* = 0.70).

### 2.3. Outcome Classification and Procedure

Children were identified as late talkers if they had a vocabulary size (word production) at or below the 10th percentile at 30 months at the Italian MB-CDI Words and Sentences Short-Form [65], a cognitive score ≥ 70 at the Italian version of the BSID-III [68] and no neurological, sensory, or motor impairments.

Children screened as late talkers on the Italian MB-CDI Words and Sentences Short-Form [65], which was filled out online by their parents, were invited with their parents to the Developmental Psychology Lab, Department of Psychology, University of Bologna, for a direct assessment of their cognitive and linguistic skills through the Italian version of the BSID-III [68]. At that same appointment, parents filled out the Italian versions of the Infant Sleep Questionnaire (ISQ) [71], the Parental Interactive Bedtime Behaviour Scale (PIBBS) [26], and the Parenting Stress Index [75].

Descriptive data concerning late talkers’ linguistic and cognitive scores are presented in Table 1. Late talkers had a mean expressive vocabulary size (*M* = 18.75, *SD* = 14.18, range = 2–43) falling below the 10th percentile, a mean language composite score falling below the normal range (*M* = 80.33, *SD* = 10.56, range = 52–106), and a mean cognitive composite score falling within the normal range (*M* = 87.00, *SD* = 7.79, range = 70–105). No significant differences were found on linguistic and cognitive scores between low-risk preterm and full-term late talkers. Descriptive data on mothers’ parenting stress index, assessed with the PSI-4 Short Form, are also reported in Table 1. Mean mothers’ parenting stress index total score was comparable with the standardized mean scores of the Italian population at the same age [75], highlighting that globally the sample of the present study was not characterized by clinical problems concerning parental distress, parent–child relationship, or child characteristics. No significant differences were found on mothers’ parenting stress index between low-risk preterm and full-term late talkers (see Table 1).

### 2.4. Statistical Analyses 

All statistical analyses were carried out using IBM SPSS Statistics 27 [77] for Windows, using a bilateral test with *p* set at <0.05. Data were checked for violation of assumptions using Kolmogorov–Smirnov test indicating that most variables were normally distributed. 

With regard to the first aim, mean and standard deviations of all variables were computed to describe perceived night sleep characteristics of late talkers, i.e., settling difficulties, night wakings, and frequency of co-sleeping as well as the bedtime practices used by their parents. Furthermore, correlational analyses were carried out to assess the associations among night sleep characteristics and parental bedtime practices in late talkers.

With regard to the second aim, hierarchical linear regression analyses were performed to investigate how individual variables (i.e., birth condition, gender, birth order), socio-demographic variables (i.e., maternal age and maternal education), and a socio-relational variable (i.e., mother’s parenting stress), were associated with perceived night sleep difficulties (i.e., infant sleep difficulties total score, settling difficulties, night wakings, co-sleeping), and parental bedtime practices (i.e., parental bedtime practices total score, active physical comforting, encouraging autonomy, leaving to cry) in late talkers. In each regression analysis, individual variables were entered first (Model 1), socio-demographic variables were added subsequently (Model 2), and the socio-relational variable was entered at the end (Model 3), as predictors of the considered dependent variables.

## 3. Results

### 3.1. Night Sleep, Parental Bedtime Practices and Their Associations in Late Talkers 

Descriptive data on perceived night sleep characteristics and parental bedtime practices in late talkers, based on parents’ responses to the ISQ and PIBBS questionnaires, are presented in Table 2 (see also Table A1 and Table A2 in the Appendix A for data concerning frequency of late talkers’ perceived night sleep characteristics and parental bedtime practices).

Regarding perceived night sleep characteristics (see Table 2), slight settling difficulties were present in the whole sample concerning both the amount of time latency needed for settling the child and the number of nights occurring per week. Moreover, a few night wakings during the week and per night with an often-short time for settling after night waking were reported. By contrast, co-sleeping was a frequent parental practice in the whole sample. Looking more specifically (see Table A1), sleep onset required less than 10 min for 12.8% (very short settling latency) and 10–30 min for 61.7% of the children (short settling latency), whereas it required 30–50 min or longer for 21.3% (long settling latency) and 4.3% (very long settling latency) of the children. Night wakings per week and per night were very rare for 36.2% and 47.8% of the children, respectively. On the other hand, night waking per week was often or very often for 12.8% and 36.2% of the children, respectively. Sleep latency after night waking was very short for 78.3% of the children, and short for most of the remaining ones (19.6%), whereas it was long only for 2.2% of the children. Co-sleeping was very rare or rare in 50% and 4.3% of the sample, respectively, whereas often or very often in 6.5% and 39.1% of the sample, respectively. Only eight (16.7%) mothers judged their child having sleeping difficulties: six of them (13%) reported mild sleep difficulties in their children, one (2.2%) moderate sleep difficulties, and one (2.2%) severe sleep difficulties. All other mothers (82.6% of the sample) did not perceive sleep difficulties in their child.

Concerning parental bedtime practices (see Table 2), encouraging autonomy practices were frequently used by parents, whereas active physical comforting practices were less frequently used, and leaving to cry was very rarely employed. Looking more specifically (see Table A2), among active physical comforting practices often or very often reported, 50% of the parents stroked or patted their child, 39.1% gave him/her a feed/drink and 39.1% settled their child in parents’ bed. Regarding encouraging autonomy practices often or very often reported, talking softly (43.5%), singing a lullaby (43.5%), reading a story (34.8%), offering a special toy/cloth (47.8%), standing near a cot (43.5%), or lying with the child next to their cot (52.2%) were used by parents. In addition, 82.6% of the parents never left their child to cry as a bedtime parenting strategy.

Bivariate Pearson correlations, carried out to investigate how perceived night sleep characteristics and parental bedtime practices were related in late talkers, are presented in Table 3. Regarding the relationships between perceived night sleep characteristics and parental bedtime practices, the ISQ total score, the night waking subscale, and co-sleeping were positively correlated with leaving to cry (respectively *r*(45) = 0.35, 0.44, and 0.31, *p* < 0.05 and <0.01). In addition, the settling difficulties subscale was positively correlated with the PIBBS total score (*r*(46) = 0.35, *p* = 0.018) and the encouraging autonomy subscale (*r*(46) = 0.38, *p* = 0.01). No other significant correlations were found.

### 3.2. Associations of Individual, Socio-Demographic and Socio-Relational Variables with Sleep Difficulties and Parental Bedtime Practices

Concerning perceived night sleep difficulties, results of hierarchical linear regression analyses described in Table 4 show that, in the first model, individual variables accounted for 28.9% of the variance of the ISQ total score with low-risk preterm birth condition having a significant contribution (Model 1: *R^2^* = 0.289, *F*(3,40) = 5.415, *p* = 0.003). In the second model, socio-demographic variables did not significantly contribute to the model and low-risk preterm birth condition remained the only significant predictor of the ISQ total score (Model 2: *R^2^* = 0.335, *F*(5,38) = 3.831, *p* = 0.007, *R^2^_change_* = 0.046, *F_change_*(2,38) = 1.324, *p* = 0.278). In Model 3, the socio-relational variable increased the accounted total variance of 21% with the level of mother’s parenting stress and low-risk preterm condition accounting for 54.5% of the total variance of the ISQ total score (Model 3: *R^2^* = 0.545, *F*(6,37) = 7.389, *p* < 0.001, *R^2^_change_* = 0.210*, F_change_*(1,37) *=* 17.076, *p* < 0.001).

Concerning settling difficulties, individual variables in Model 1 did not contribute to predict settling difficulties (Model 1: *R^2^* = 0.121, *F*(3,40) = 1.89, *p* = 0.147). In Model 2, being first born, among individual variables, and high mother’s educational level, among socio-demographic variables, explained the 25.2% of the variance with a significant increase from Model 1 (*R^2^* = 0.252, *F*(5,38) = 2.625, *p* = 0.039, *R^2^_change_* = 0.130, *F_change_*(2,38) = 3.401, *p* = 0.044). The socio-relational variable significantly increased the variance of settling difficulties by 12.1% (Model 3) with the level of stress perceived by mothers, being first born, and high maternal educational level accounting for the 37.3% of the total variance (Model 3: *R^2^* = 0.373, *F*(6,37) = 3.764, *p* = 0.005; *R^2^_change_* = 0.121, *F_change_*(1,37) = 7.329, *p* = 0.010).

Concerning night waking, individual variables accounted for the 21.8% of the total variance with low-risk preterm birth condition having a significant contribution (Model 1: *R^2^* = 0.218, *F*(3,40) = 3.706, *p* = 0.019). In the second model, socio-demographic variables did not significantly increase the explained variance (Model 2: *R^2^* = 0.226, *F*(5,38) = 2.222, *p* = 0.072, *R^2^_change_* = 0.009*, F_change_*(2,38) = 0.214, *p* = 0.808). By contrast, the socio-relational variable increased the explained variance by 12.2% (Model 3) with the level of stress perceived by mothers and low-risk preterm condition accounting for the 34.8% of the total variance (Model 3: *R^2^* = 0.348, *F*(6,37) = 3.295, *p* = 0.011, *R^2^_change_* = 0.122, *F_change_*(1,37) = 6.925, *p* = 0.012).

With regard to co-sleeping, individual variables accounted for the 19.9% of the total variance with low-risk preterm birth condition having a significant contribution (Model 1: *R^2^* = 0.199, *F*(3,40) = 3.317, *p* = 0.029). In the second model, socio-demographic variables did not significantly contribute to the model and low-risk preterm birth condition remained the only significant predictor of co-sleeping (Model 2: *R^2^* = 0.271, *F*(5,38) = 2.827, *p* = 0.029; *R^2^_change_* = 0.072*, F_change_*(2,38) = 1.875, *p* = 0.167). The socio-relational variable increased the explained variance of co-sleeping by 10.3% (Model 3) with the level of stress perceived by mothers, but no longer the low-risk preterm condition, accounting for the 37.4% of the total variance (Model 3: *R^2^* = 0.374, *F*(6,37) = 3.681, *p* = 0.006; *R^2^_change_* = 0.103*, F_change_*(1,37) = 6.065, *p* = 0.019).

Concerning parental bedtime practices, results of the hierarchical linear regression analyses displayed in Table 5, showed no significant associations between parental bedtime practices and individual, socio-demographic and socio-relational variables except for leaving to cry. In the first model, individual variables, did not predict leaving to cry (Model 1: *R^2^* = 0.052, *F*(3,40) = 0.737, *p* = 0.536). In the second model, high mother’s educational level negatively predicted leaving to cry, accounting for the 21.6% of the total variance and increasing the explained variance by 16.3% (Model 2: *R^2^* = 0.216, *F*(5,38) = 2.089, *p* = 0.088; *R^2^_change_* = 0.163*, F_change_*(2,38) = 3.955, *p* = 0.028). The socio-relational variable increased the explained variance by 15.4% (Model 3) with the level of stress perceived by mothers, but no longer high mother’s educational level, accounting for the 36.9% of the total variance (Model 3: *R^2^* = 0.369, *F*(6,37) = 3.609, *p* = 0.006; *R^2^_change_* = 0.154*, F_change_*(1,37) *=* 9.005, *p* = 0.005).

## 4. Discussion

The present study brought innovative findings about perceived night sleep characteristics, parental bedtime practices, their relationships, and associated individual, socio-demographic, and socio-relational factors in 30-month-old late talkers, a population about which very little knowledge is available in the literature regarding these issues.

### 4.1. Perceived Night Sleep Characteristics, Parental Bedtime Practices, and Their Associations in Late Talkers

In this study, we wanted to describe perceived night sleep characteristics (i.e., settling difficulties, night wakings, and co-sleeping) and parental bedtime practices (i.e., active physical comforting, encouraging autonomy, and leaving to cry) by using questionnaires filled out by parents (i.e., the ISQ and the PIBBS).

Concerning child night sleep, high latency in falling asleep and the number of night wakings are two of the main characteristics that lead parents to consider their child’s sleep problematic [9]. Our findings suggest that among late talkers, more than half did not usually have difficulties in settling at night. However, about one third had frequent night wakings. Results obtained by Blair et al. [43], whose participants were English families from the general population, showed that 50% of the parents reported that their 30-month-old children never woke up during night sleep, 44% of parents reported that their child usually woke up once or twice a night, and 5% reported more than three night wakings. Further research should compare late talkers with same-aged typically developing children belonging to the same culture for further examining whether night waking is more frequent in late talkers than typically developing children.

The ISQ questionnaire also allowed us to analyze the frequency of co-sleeping in late talkers, which is a frequent sleeping strategy depending on multiple factors [25,29,58,78]. Co-sleeping was often or very often employed by more than 40% of the parents of our sample, whereas half of the parents (50%) reported that their child never slept with them or did so less than once a week. In the work of Sadeh et al. [9], 15% of the parents of children aged 24–34 months reported bringing their child most of the time into their bed during the night as a sleep initiation method and 20.71% of parents as a resuming sleep method. However, participants of that study were from the US and Canada and these results are not completely comparable with ours as co-sleeping is a strictly culture-dependent bedtime method [42]. Future research with 30-month-old typically developing children is needed to compare our results.

Regarding bedtime practices, in accordance with our hypothesis, results showed that parental bedtime practices based on encouraging autonomy were frequently used, whereas active physical comforting ones were less frequently employed, as has been observed in typically developing children of the same age belonging to Western cultures (e.g., North American culture) [9]. Moreover, concerning letting the child cry, as a parental bedtime practice, without any kind of intervention, more than 80% of the parents in the sample reported never using this strategy. This result was not in line with the findings of Morrell and Cortina-Borja [26] as only 28% of the parents in their sample did not let their child cry as a bedtime practice. This difference could have several explanations. First, the children in the sample of Morrel and Cortina-Borja’s study, being between 12 and 19 months old, were younger than those in the current study. Second, they belonged to the English culture that, although a Western culture, is different from the Italian one of the current study. Indeed, parental bedtime practices vary among cultures [42,57,59,79] and ignoring children’s bedtime crying is a strategy used in some behavioral intervention programs (e.g., Extinction-Based Behavioral Sleep Interventions) applied in Western countries [80]. However, Schwichtenberg et al. [81] affirmed that different beliefs and parenting behaviors regarding child night sleep might be present within Western countries. For this reason, we can hypothesize that Italian parents’ cultural sleep-related beliefs lead them to be less in favor of ignoring their child’s crying during bedtime. For many parents, this settling behavior can be too stressful and difficult to implement, because it may collide with their beliefs about setting limits to resist the child’s demands and because it can be considered inappropriate and negligent of the child’s needs [82,83,84,85]. Therefore, we can affirm that leaving a child of 30 months to cry is not a frequent practice used by parents at this age in the Italian culture, at least with late talking children.

We also aimed to describe how perceived night sleep characteristics and parental bedtime practices were related to each other in late talkers. The main findings showed that recurrent settling difficulties were reported by parents who often applied bedtime practices and, in particular, encouraging autonomy practices. Previous research observed that night sleep, compared to daytime sleep, can be more influenced by environmental factors, including parental bedtime practices, emphasizing that regularity and type of parental bedtime practices were good predictors of sleep quality [9,25,26]. Encouraging autonomy practices were positively linked to better night sleep quality in the study by Morrell and Cortina-Borja [26], but the results of the present study showed an opposite relationship with respect to settling. These results may have several explanations that should be investigated in further studies. A bidirectional influence between children’s perceived night sleep characteristics and bedtime practices should be considered, as perceived sleep difficulties can be affected by parental interactive behaviors and can, in turn, cause increased stress levels in parents [25] as we will describe in the following paragraph. For this reason, parents who reported more difficulties in their child falling asleep may more often use bedtime practices with the intent of sleep facilitation. At the same time, excessively frequent use of bedtime practices in 30-month-old late talkers could be the cause of settling difficulties as perceived by parents. Indeed, Morrell [83] highlighted how an over-invasive bedtime behavior, due to problematic parental beliefs about settling limits, could be linked to the emergence and maintenance of sleep problems. As in our sample, the usage of parental bedtime practices was moderate but not too frequent. Hence, it may be more plausible that the significant relation between encouraging autonomy practices and settling difficulties was due to the fact that encouraging autonomy practices were frequently used.

Furthermore, the number of night wakings was positively related to the frequency of co-sleeping. This result is in line with Volkovich and colleagues’ study [79], which identified through sleep diaries a greater number of night wakings in children who slept with their parents at six months than in children who slept alone, although this finding was not matched by measurements obtained by actigraphy. Therefore, our findings might be explained by the fact that parents who slept with their children perceived more night wakings compared with those whose children slept alone. It might be interesting to further verify this finding by applying objective measurements in 30-month-old late talkers in future studies.

### 4.2. Association of Individual, Socio-Demographic, and Socio-Relational Variables with Perceived Sleep Difficulties and Bedtime Parental Practices in Late Talkers

The second aim of this study was to investigate how individual, socio-demographic, and socio-relational variables (i.e., birth condition, gender, birth order, maternal age, maternal education level, and mother’s parenting stress) were associated with perceived sleep difficulties and parental bedtime practices in late talkers.

As described in Section 1, several studies exploring individual and environmental factors linked to sleep characteristics and parental bedtime practices have been conducted mostly on typically developing children, with some on atypically developing children [9,10,13,33,43,48,49,50,51,52,53,55,56,86], but not on late talkers. The present study provides new findings on a sample of 30-month-old late talkers.

Concerning perceived night sleep difficulties, results showed that low-risk preterm birth condition and mother’s parenting stress explained a portion of the variance in the ISQ total score and night waking subscale score. First-born condition, low maternal education, and mother’s parenting stress predicted settling difficulties scores. In addition, mother’s parenting stress was the only factor predicting co-sleeping frequency. These results were only partially expected. 

Regarding preterm birth, evidence in the literature about sleep characteristics of these infants is discordant: some studies have found no substantial differences with full-term born infants [12,46], whereas others highlighted some differences, with more altered sleep patterns in children with higher neonatal immaturity [44,45]. The present study’s results are in line with this latter group of researchers, showing that even a low-risk preterm birth can more frequently contribute to fragmented night sleep patterns in late talkers. Thus, preterm birth, even when low-risk, appears to be a risk factor for night sleep organization.

Mother’s parenting stress, regardless of birth condition, was associated with difficulties in falling asleep and night wakings, confirming the findings of previous studies [51,55]. Asaka and Takada [44] also found that the presence of more disrupted night sleep reported by parents of preterm versus full-term infants was related to the fact that their mothers were much more concerned about their infants’ sleep problems. For this reason, mother’s parenting stress together with low-risk preterm birth condition can predict reported sleep problems, and this is supported by more frequent night wakings detected by parents. Except for low-risk preterm birth condition, none of the other biological and socio-demographic factors predicted ISQ total score and night waking score. By contrast, being first-born and having a mother with a high educational level predicted having more settling difficulties. These findings are only partially in line with previous research. In Blair et al. [43] study, conducted on the general population, maternal education was not associated with total sleep duration. In addition, McDonald et al. [48] found opposite results to ours: lower maternal education increased the odds of shorter sleep. Concerning the relationship between birth order and sleep patterns, our findings extended to late talkers the evidence found in the general population by Plancoulaine et al. [13], who highlighted how being a first-born child is a risk factor for shorter sleep duration, stating that it might be explained by higher parental stress or higher parental bedtime intervention or both. Given that studies investigating the relationship between sleep patterns and biological and socio-demographic factors are mainly concerned with sleep durations and night wakings, but not with settling difficulties and given the presence of non-concordant results in previous studies, further research is needed to explore and explain these issues in late talkers.

Mother’s parenting stress was also associated with frequency of co-sleeping. This finding could be interpreted in a bidirectional way. On the one hand, co-sleeping in Western societies is considered a problem [58,87]; its use can cause more stress in parents, also because those that frequently sleep with their child have more sleep problems than parents of non-co-sleeping infants [79]. On the other hand, an open debate on the physical and psychological risks and benefits of co-sleeping is still ongoing. Our findings showed that mother’s parenting stress increased with co-sleeping, highlighting a positive association between them, differently from other parental bedtime practices that reduced parenting stress and ameliorated family functioning [33]. It might also be that parents who are most concerned about their child’s problems slept with them to reduce their own distress and better regulate and control them, as maintained b y Mckenna et al. [88].

Regarding parental bedtime practices, results showed that neither biological, nor socio-demographic, nor socio-relational variables were related to behaviors used by parents of late talkers for trying to settle them. These findings did not support our assumption regarding the existence of associations between the use of parental bedtime practices and these factors. These results were also not in line with previous research. Concerning birth condition, an explanation could be the fact that preterm children were characterized by low perinatal risk. As preterm children participating in the current study did not have severe neonatal complications, unlike high-risk preterm children, their parents might not have felt the need to frequently intervene to help them falling asleep. Relations between parental bedtime practices and individual, socio-demographic, and socio-relational variables in late talkers should be further explored in future research, especially regarding their links to parenting stress, which is a fundamental element affecting the parent–child relationship at bedtime.

### 4.3. Limitations and Future Directions

Although this study brings innovative findings about perceived sleep characteristics and parental bedtime practices in late talkers, some limitations should be considered.

First, the use of subjective measures such as parental questionnaires could lead to bias in the results due to several factors [89,90]. Among these, we can consider children’s biological characteristics: parents of children with medical problems or developmental impairments may underestimate their child’s sleep problems, because they are focused on other problematic conditions or because they do not expect their child to “sleep through the night” as much [16,91]. Perceived sleep difficulties may also not be predicted mainly by parental socio-demographic characteristics, i.e., socio-economic status, educational level, age, and culture [9,92], but rather by parental beliefs regarding setting limits, that is the attempt to resist to the child’s demands, child health worries, parental stress, and doubt regarding being a good parent [83,90]. Interestingly, Gossé and colleagues [90] found that there was a better agreement between objective and subjective measures on night waking parameters in children whose mothers reported higher stress and anxiety levels. Moreover, parental bedtime practices can affect the outcomes of subjective measurements: mothers of co-sleeping children reported a greater number of night wakings than mothers whose children slept alone, but in previous studies this difference was not matched by measurements obtained by actigraphy [79]. Further research is thus necessary to investigate sleep patterns and parental bedtime practices in late talkers by complementing the use of parental questionnaires, i.e., ISQ and PIBBS, with objective measurements such as actigraphy to record children’s sleep characteristics faithfully, non-invasively, and without subjective bias.

Second, the absence of a group of typically developing children does not make it possible to determine whether late talkers exhibited any distinctive sleep characteristics regarding difficulties in falling asleep (i.e., frequency of settling difficulties, night wakings, and co-sleeping) and parental bedtime practices. A future comparison of late talkers with same-age and same-culture typically developing children would allow to understand whether they differ in any specific perceived sleep characteristic and parental bedtime strategy.

Third, the inclusion in the current sample of late talkers born at term or preterm with low perinatal risk, does not allow generalizing our findings to all late talkers born preterm. Children born preterm with a higher perinatal risk might have more severe complications and alterations in sleep patterns [44]. Thus, further studies should be conducted on late talkers, including children born preterm with higher perinatal risk conditions and higher neonatal immaturity.

Fourth, in the present work, all measurements were taken at a single age. Thus, it was not possible to monitor changes in sleep characteristics and parental bedtime practices and their relations over time in late talkers. Further longitudinal studies are needed to face this issue.

### 4.4. Clinical Implications and Conclusions

Identifying risk factors for the emergence of perceived sleep difficulties in late talkers and specific parental bedtime practices used with these children would allow for the early monitoring, planning, and implementation of intervention programs in at-risk populations to promote optimal child sleep patterns. Interventions should be directed to the child and the environment surrounding her/him, by coaching parents on the unique characteristics of sleep at different ages while considering individual, social, and cultural variability that could shape alternative practices to apply at bedtime. This would promote better child health as well as enhance optimal cognitive, language, behavioral, socio-relational, and motor development [93].

## Figures and Tables

**Table 1:** Individual, Socio-demographic and Socio-Relational Characteristics, Language and Cognitive Scores of All, Low-Risk Preterm, and Full-Term Late Talkers.

	All Late Talkers (*n* = 47)			Low-Risk Preterm Late Talkers (*n* = 24)			Full-term Late Talkers (*n* = 23)				
Late Talkers’ Characteristics	*M/n*	*SD/%*	*Range*	*M/n*	*SD/%*	*Range*	*M/n*	*SD/%*	*Range*	*t/*χ^2^	*p*
Gestational Age (weeks), *M, SD*	36.50	3.47	30–41.29	33.55	2.06	30–36.86	39.58	1.16	37–41.29	12.30	**<0.001**
Birthweight (grams), *M, SD*	2631	864	1080–4040	1877	408	1080–2620	3417	348	2850–4040	13.90	**<0.001**
Gender (male), *n,%*	39	83.0		20	83.3		19	82.6			1.000 ^
Firstborn, *n,%*	25	53.2		13	54.2		12	52.2		0.019	1.000 ^§^
Twins, *n, %*	14	29.8		14	58.3		0	0		19.11	**<0.001** ^§^
Type of Delivery (caesarean), *n, %*	22	46.8		20	83.3		2	8.7		26.28	**<0.001** ^§^
Length of Stay in Hospital (days), *M, SD*	12.15	17.67	0–78	21.58	20.82	2–78	2.30	1.19	0–7	−4.43	**<0.001**
Otitis Media (> 4 episodes/year), *n, %*	2	4.3		1	4.2		1	4.3			1.000 ^
Family History of Language/Learning Disorders, *n, %*	6	12.8		2	8.3		4	17.4			0.416 ^
Child Care Center Attendance, *n, %*	35	74.5		16	66.7		19	82.6		1.57	0.318 ^§^
Exposure to Another Language, *n, %*	8	17.0		6	25.0		2	8.7			0.245 ^
Mother’s Age (years), *M, SD*	38.22	5.76	25–49	38.83	5.75	30–49	37.55	5.84	25–47	−0.75	0.455
Father’s Age (years), *M, SD*	40.00	6.15	29–52	40.58	6.40	29–52	39.36	5.95	29–51	−0.67	0.508
Mothers with High Educational Level (>13 y), *n, %*	32	68.1		15	62.5		17	73.9		0.70	0.534 ^§^
Fathers with High Educational Level (>13 y), *n, %*	22	46.8		8	33.3		14	60.9		3.58	0.082 ^§^
Mother’s Nationality (Italian), *n, %*	40	85.1		19	79.2		21	91.3			0.416 ^
Father’s Nationality (Italian), *n, %*	41	87.2		19	79.2		22	95.7			0.188 ^
Mother’s Parenting Stress Index Total Score, *M*, *SD*	66.76	18.06	39–113	69.65	18.08	40–109	63.73	17.95	39–113	−1.10	0.276
*Language and Cognitive Scores*											
MB-CDI Word Production, *M*, *SD*	18.75	14.18	2–43	21.50	15.22	2–43	15.87	12.70	2–40	−1.37	0.176
Bayley-III Language Composite Score, *M*, *SD*	80.33	10.56	52–106	81.38	7.41	66–100	79.16	13.33	52–106	−0.64	0.526
Bayley-III Cognitive Composite Score, *M*, *SD*	87.00	7.79	70–105	88.13	6.89	75–105	85.71	8.70	70–100	−1.04	0.306
Age of Assessment (months), *M*, *SD*	31.33	1.49	27.20–34.26	31.52	1.39	29.10–34.26	31.13	1.60	27.20–33.89	−0.92	0.365

Note. Significant results concerning the comparison between low-risk preterm children and full-term children are reported in bold for the Chi-square test (^§^) or the Fisher’s exact test (^) when at least one expected value was <5.0. Missing data: Mother’s age, *n* = 1; Father’s age, *n* = 1; Cognitive composite score, *n* = 2; Language composite score, *n* = 7; Mother’s Parenting Stress Index Total Score, *n* = 2.

**Table 2:** Late Talkers’ Perceived Night Sleep Characteristics Assessed with the Infant Sleep Questionnaire (ISQ) and Parental Bedtime Practices Assessed with the Parental Interactive Bedtime Behaviour Scale (PIBBS).

	Late Talkers (*n* = 47)		
	*M/n*	*SD/%*	*Range*
ISQ Total score	9.91	7.43	1–32
Settling Difficulties	3.19	3.30	0–12
Settling Latency	1.85	1.43	0–6
Settling Difficulties per Week	1.34	2.15	0–7
Night Waking	3.93	3.83	0–14
Night Waking per Week	3.02	2.94	0–7
Night Waking per Night	0.72	0.89	0–4
Sleep Latency after Night Waking	0.28	0.62	0–3
Co-sleeping	2.96	3.24	0–7
Maternal Perception of Sleep Difficulties	0.24	0.60	0–3
PIBBS Total Score	22.20	9.94	4–48
Active Physical Comforting	8.91	5.73	0–28
Stroking or Patting	2.17	1.60	0–4
Cuddling or Rocking in Arms	0.89	1.37	0–4
Carrying around House in Arms	0.43	1.09	0–4
Walking in Pram or Buggy	0.13	0.45	0–2
Car Ridings	0.13	0.54	0–3
Playing with Child	0.70	1.07	0–4
Giving a Feed/Drink	1.80	1.56	0–4
Settling on Sofa with Parent	0.80	1.22	0–4
Settling in Parent’s Bed	1.85	1.66	0–4
Encouraging Autonomy	12.89	6.53	0–24
Music Tape or Musical Toy	0.93	1.41	0–4
Talking Softly to Child	2.00	1.52	0–4
Singing a Lullaby	2.00	1.66	0–4
Reading a Story to Child	1.96	1.38	0–4
Offering a Special Toy/Cloth	1.98	1.75	0–4
Standing near Cot without Picking Baby Up	1.83	1.72	0–4
Lying with Child next to their Cot	2.20	1.72	0–4
Leaving to Cry	0.39	0.93	0–3

Note. Missing data: ISQ (Total score), *n* = 1; Night Waking, *n* = 1; Co-sleeping, *n* = 1; PIBBS (Total score), *n* = 1; Active Physical Comforting, *n* = 1; Encouraging Autonomy, *n* = 1; Leaving to Cry, *n* = 1.

**Table 3:** Correlations between Late Talkers’ Perceived Night Sleep Characteristics and Parental Bedtime Practices.

Pearsons’ Correlations	PIBBS Total Score	Active Physical Comforting	Encouraging Autonomy	Leaving to Cry
ISQ Total Score	0.23	0.20	0.13	0.35 *
Settling Difficulties	0.35 *	0.18	0.38 **	−0.04
Night Waking	0.13	0.08	0.06	0.44 **
Co-sleeping	0.01	0.17	−0.19	0.31 *

Note. Missing data: ISQ Total score, *n* = 1; Night Waking, *n* = 1; Co-sleeping, *n* = 1; PIBBS total score, *n* = 1; Active Physical Comforting, *n* = 1; Encouraging Autonomy, *n* = 1; Leaving to cry, *n* = 1. * *p* < 0.05, ** *p* < 0.01.

**Table 4:** Hierarchical Regression Analysis for ISQ Total Score, Settling difficulties, Night Waking and Co-sleeping.

	Dependent Variables							
Independent Variables	ISQ Total Score		Settling Difficulties		Night Waking		Co-Sleeping	
	*B*	*β*	*B*	*β*	*B*	*β*	*B*	*β*
Model 1								
Low-Risk Preterm Birth	5.69 **	0.39	0.71	0.11	2.57 *	0.34	2.04 *	0.32
Male Gender	4.38	0.23	−0.18	−0.02	2.75	0.28	1.97	0.24
Firstborn	3.48	0.24	2.09 *	0.33	0.82	0.11	0.91	0.14
*R^2^*	0.289 **		0.121		0.218 *		0.199 *	
Model 2								
Low-Risk Preterm Birth	5.54 **	0.38	0.95	0.15	2.51 *	0.33	1.80 *	0.28
Male Gender	3.66	0.19	−0.47	−0.06	2.59	0.26	1.71	0.21
Firstborn	3.72	0.26	2.61 **	0.41	0.83	0.11	0.65	0.10
High Mother’s Educational Level	1.18	0.08	2.44 *	0.36	0.10	0.01	−1.04	−0.16
Mother’s Age	0.25	0.20	0.03	0.05	0.06	0.09	0.14	0.25
*R^2^*	0.335 **		0.252 *		0.226		0.271 *	
*R^2^* *Change*	0.046		0.130 *		0.009		0.072	
Model 3								
Low-Risk Preterm Birth	4.61 **	0.32	0.68	0.11	2.14 *	0.28	1.51	0.24
Male Gender	3.22	0.17	−0.63	−0.08	2.41	0.25	1.58	0.19
Firstborn	3.29	0.23	2.42 **	0.38	0.66	0.09	0.52	0.08
High Mother’s Educational Level	2.84	0.19	2.95 **	0.44	0.76	0.09	−0.54	−0.08
Mother’s Age	0.11	0.09	−0.01	−0.02	0.01	0.01	0.10	0.18
Mother’s Parenting Stress	0.20 ***	0.49	0.07 *	0.37	0.08 *	0.37	0.06 *	0.34
*R^2^*	0.545 ***		0.373 **		0.348 *		0.374 **	
*R^2^* *Change*	0.210 ***		0.121 *		0.122 *		0.103 *	

Note. In the hierarchical regression analysis, individual variables (low-risk preterm birth, male gender, and firstborn) were entered first (Model 1); environmental variables (high mothers’ educational level i.e., >13 years, and mother’s age) were subsequently added (Model 2); the socio-relational variable (PSI total score) was finally entered (Model 3). * *p* < 0.05; ** *p* < 0.01; *** *p* < 0.001.

**Table 5:** Hierarchical Regression Analysis for PIBBS Total Score, Active Physical Comforting, Encouraging Autonomy, and Leaving to Cry.

	Dependent Variables							
Independent Variables	PIBBS Total Score		Active Physical Comforting		Encouraging Autonomy		Leaving to Cry	
	*B*	*β*	*B*	*β*	*B*	*β*	*B*	*β*
Model 1								
Low-Risk Preterm Birth	0.73	0.04	2.09	0.19	−1.55	−0.12	0.18	0.10
Male Gender	1.86	0.07	1.54	0.11	−0.17	−0.01	0.49	0.20
Firstborn	2.20	0.11	1.86	0.17	0.29	0.02	0.05	0.03
*R^2^*	0.020		0.076		0.014		0.052	
Model 2								
Low-Risk Preterm Birth	0.84	0.04	1.85	0.16	−1.13	−0.09	0.13	0.07
Male Gender	1.87	0.07	1.72	0.12	−0.47	−0.03	0.61	0.25
Firstborn	2.35	0.12	1.40	0.12	1.05	0.08	−0.10	−0.06
High Mother’s Educational Level	0.85	0.04	−2.46	−0.20	4.07	0.29	−0.76 *	−0.38
Mother’s Age	−0.04	−0.02	0.02	0.02	−0.04	−0.04	−0.02	−0.12
*R^2^*	0.022		0.114		0.091		0.216	
*R^2^* *Change*	0.002		0.038		0.077		0.163 *	
Model 3								
Low-Risk Preterm Birth	0.62	0.03	1.78	0.16	−1.18	−0.09	0.03	0.01
Male Gender	1.74	0.07	1.68	0.12	−0.50	−0.03	0.55	0.23
Firstborn	2.19	0.11	1.35	0.12	1.02	0.08	−0.18	−0.10
High Mother’s Educational Level	1.26	0.06	−2.34	−0.19	4.16	0.30	−0.56	−0.28
Mother’s Age	−0.07	−0.04	0.01	0.01	−0.05	−0.04	−0.04	−0.22
Mother’s Parenting Stress	0.05	0.08	0.01	0.04	0.01	0.03	0.02 **	0.42
*R^2^*	0.028		0.116		0.092		0.369 **	
*R^2^* *Change*	0.006		0.002		0.001		0.154 **	

Note. In the hierarchical regression analysis, individual variables (low-risk preterm birth, male gender and firstborn) were entered first (Model 1); environmental variables (high mothers’ educational level i.e., >13 years, and mother’s age) were subsequently added (Model 2); the socio-relational variable (PSI total score) was finally entered (Model 3). * *p* < 0.05; ** *p* < 0.01.

## Data Availability

The dataset presented in this article is not readily available because it includes sensitive information about minors with developmental vulnerabilities. Requests to access the dataset should be directed to corresponding authors.

## Appendix A

**Table A1:** Frequency of Late Talkers’ Perceived Nighttime Sleep Characteristics Assessed with the Infant Sleep Questionnaire (ISQ).

	Very Short/ Very Rarely		Short/ Sometimes		Long/ Often		Very Long/ Very Often	
ISQ	*N*	*%*	*N*	*%*	*N*	*%*	*N*	*%*
Settling								
Settling Latency	6	12.8	29	61.7	10	21.3	2	4.3
Settling Difficulties per Week	29	61.7	8	17	5	10.6	5	10.6
Night Waking								
Night Waking per Week	17	36.2	7	14.9	6	12.8	17	36.2
Night Waking per Night	22	47.8	22	47.8	2	4.3	0	0
Sleep Latency after Night Waking	36	78.3	9	19.6	1	2.2	0	0
Co-sleeping	23	50.0	2	4.3	3	6.5	18	39.1
	**Absent**		**Mild**		**Moderate**		**Severe**	
Sleep Difficulties (Maternal Criterion)	38	82.6	6	13.0	1	2.2	1	2.2

Note. Missing data: Night Waking per night, *n* = 1; Sleep Latency after Night Waking, *n* = 1; Co-sleeping, *n* = 1; Sleep difficulties (maternal criterion), *n* = 1. Very Short/Very Rarely corresponds to point 0, Short/Sometimes corresponds to point 1 or 2, Long/Often corresponds to point 3 or 4, Very Long/Very Often corresponds to point 5 or 6 or 7. Absent corresponds to point 0, Mild to point 1, Moderate to point 2, Severe to point 3.

**Table A2:** Frequency of Parental Bedtime Practices Assessed with the Parental Interactive Bedtime Behaviour Scale (PIBBS).

	Never		Rarely/ Sometimes		Often/ Very Often	
PIBBS	*N*	*%*	*N*	*%*	*N*	*%*
Active Physical Comforting						
Stroking or Patting	13	28.3	10	21.7	23	50.0
Cuddling or Rocking in Arms	29	63.0	11	23.9	6	13.0
Carrying around House in Arms	37	80.4	5	10.9	4	8.7
Walking in Pram or Buggy	42	91.3	4	8.7	0	0
Car Riding	43	93.5	2	4.3	1	2.2
Playing with Child	28	60.9	15	32.6	3	6.5
Giving a Feed/Drink	17	37.0	11	23.9	18	39.1
Settling on Sofa with Parent	29	63.0	11	23.9	6	13.0
Settling in Parent’s Bed	15	32.6	13	28.3	18	39.1
Encouraging Autonomy						
Music Tape or Musical Toy	29	63.0	8	17.4	9	19.6
Talking Softly to Child	12	26.1	14	30.4	20	43.5
Singing a Lullaby	16	34.8	10	21.7	20	43.5
Reading a Story to Child	10	21.7	20	43.5	16	34.8
Offering a Special Toy/Cloth	18	39.1	6	13.0	22	47.8
Standing near Cot without Picking Baby Up	17	37.0	9	19.6	20	43.5
Lying with Child next to their Cot	14	30.4	8	17.4	24	52.2
Leaving to Cry	38	82.6	4	8.7	4	8.7

Note. Missing data: Stroking part of child or patting, *n* = 1; Cuddling or rocking in arms, *n* = 1; Carrying around house in arms, *n* = 1; Walking in pram or buggy, *n* = 1; Car riding, *n* = 1; Playing with child, *n* = 1; Giving a feed/drink, *n* = 1; Settling on sofa with parent, *n* = 1; Settling in parent’s bed, *n* = 1; Music tape or musical toy, *n* = 1; Talking softly to child, *n* = 1; Singing a lullaby, *n* = 1; Reading a story to child, *n* = 1; Offering a special toy/cloth, *n* = 1; Standing near cot without picking baby up, *n* = 1; Lying with child next to their cot, *n* = 1; Leaving to cry, *n* = 1. Never corresponds to point 0, Rarely/Sometimes corresponds to point 1 or 2, Often/Very Often corresponds to point 3 or 4.
